# Reporting and representation of underserved groups in intervention studies for patients with multiple long-term conditions: a systematic review

**DOI:** 10.1177/01410768241233109

**Published:** 2024-04-16

**Authors:** Zara Kayani, Andrew Willis, Shukrat O Salisu-Olatunji, Shavez Jeffers, Kamlesh Khunti, Ash Routen

**Affiliations:** 1 Diabetes Research Centre, 4488 Leicester General Hospital, University of Leicester, Leicester LE1 7RH, UK; 2 HRB Clinical Research Facility & School of Public Health, 8795 University College Cork T12 WE28, Ireland; 3 NIHR Applied Research Collaboration–East Midlands, University of Leicester, Leicester LE5 4PW, UK

**Keywords:** Ethnic studies, evidence-based practice, public health, quantitative research, statistics and research methods

## Abstract

**Objectives:**

Globally, there is a growing number of people who are living with multiple long-term conditions (MLTCs). Due to complex management needs, it is imperative that research consists of participants who may benefit most from interventions. It is well documented that ethnic minority groups and lower socioeconomic status (SES) groups are at an increased risk of developing MLTCs. Therefore, the aim of this systematic review was to determine the level of reporting and representation of underserved groups (ethnic minority and low SES) in intervention studies addressing MLTCs.

**Design:**

Systematic review. Four databases including Cochrane Library, MEDLINE, CINAHL and Scopus were searched for intervention studies from North America or Europe published between January 1990 and July 2023.

**Setting:**

Hospital and community-based interventions. We included interventional studies focusing on improving MLTC-related outcomes.

**Participants:**

Patients with MLTCs.

**Main outcome measures:**

Total number of studies reporting on ethnicity and SES. Number and proportion of studies reporting by ethnic/SES group.

**Results:**

Thirteen studies met the inclusion criteria. Only 4 of 13 studies (31%) recorded and reported ethnicity information. Of these four studies that reported on ethnicity, three studies consisted of primarily White participants. Ethnic minority groups were underrepresented, but one study included a majority of African American participants. Moreover, 12 of 13 studies (92%) reported on SES with income and educational level being the primary measures used. SES representation of higher deprivation groups was varied due to limited data.

**Conclusions:**

For ethnicity, there was a lack of reporting, and ethnic minority groups were underrepresented in intervention studies. For SES, there was a high level of reporting but the proportion of study samples from across the spectrum of SES varied due to the variety of SES measures used. Findings highlight a need to improve the reporting and representation of ethnic minority groups and provide more detailed information for SES through using consistent measures (e.g. education, income and employment) to accurately determine the distribution of SES groups in intervention studies of people with MLTCs.

## Introduction

The development of multiple chronic diseases has become widely prevalent as a result of an ageing population, lifestyle changes and other social determinants.^1,2^ In England alone, it is estimated that one in four adults have two or more long-term health conditions,^3,4^ known as multiple long-term conditions (MLTCs, also termed multimorbidity). MLTCs have implications for both the individual and healthcare services. Having MLTCs has been linked to reduced quality of life,^5^ and an increase in adverse health outcomes including premature mortality.^6^ For healthcare services, MLTCs are a significant burden in terms of expenditure, hospital admissions and medication prescriptions.^7^

In some population groups, typically described as ‘underserved’, the burden associated with MLTCs is higher.^8^ ‘Underserved’ is a term used to refer to ‘populations that are underrepresented or disengaged from medical research or services despite a disproportionately high healthcare burden’.^9^ This may include those who are socioeconomically deprived, elderly people and women.^10^ In particular, underserved groups are often underrepresented in public health and medical research.^10^ Reasons for this could include choices on study design and recruitment strategy, such as employing restrictive inclusion criteria (e.g. participants must be able to speak English), which can disproportionally impact participation from underserved groups.^11^ Low representation of underserved groups within study samples limits the ability to discern if a treatment or intervention will benefit a given underserved group, and the generalisability of trial findings across the general population is also reduced.

Ethnic minority groups are typically underserved in health and care research. This is noteworthy as there are disparities between different ethnic groups in relation to having MLTCs.^12^ Ethnic minority groups have a greater burden of MLTCs compared with their White counterparts.^13,14^ Recent research has established that the prevalence of MLTCs is lower in those of Black, Mixed and Other ethnicities and only slightly higher in Asian ethnicities after adjusting for age and socioeconomic status (SES).^15^ However, intersectionality should be considered when understanding health inequalities. Ethnic minority groups are more likely to experience higher levels of socioeconomic deprivation.^16^ Subsequently, research has found that belonging to an ethnic group, coupled with having low SES, increases the chances of having early-onset MLTCs.^17^

SES, which is often measured through an individual’s family income, educational attainment, occupation or postcode, is another key factor that influences the development and prevalence of MLTCs. For individuals living in deprived areas, the onset of MLTCs occurred 10–15 years earlier.^18^ A study reported that the odds of MLTCs was 42% higher for participants residing in the most versus the least deprived areas (odds ratio [OR]: 1.42, 95% confidence interval [CI]: 1.41–1.42).^19^ Despite low SES groups having a greater burden of living with MLTCs,^20,21^ it is unclear as to how well deprived groups are represented in intervention studies for the management of MLTCs.

Therefore, this systematic review aims to address the following question: What is the level of reporting and representation of underserved groups (ethnic minorities and low SES) in intervention studies for people with MLTCs?

## Methods

This systematic review was registered on PROSPERO (CRD42023347900) and was prepared in accordance with PRISMA (2020) guidelines (Preferred Reporting Items for Systematic Reviews and Meta-Analyses guidelines).^22^ PRISMA checklist can be found in the supplementary materials, Appendix 1.

### Search strategy and selection criteria

Database searches for literature were conducted in Cochrane Library, MEDLINE, CINAHL and Scopus. Reference lists from relevant systematic reviews were also hand searched for any additional studies.^23,24^ Searches focused on key terms such as ‘multiple long-term conditions’, ‘multimorbidity’ and ‘interventions’. The search strategy used is detailed in the supplementary materials, Appendix 2. Searches were run from January 1990 to July 2023. The search criteria were limited to English language articles only.

Studies were included if participants were adults (aged 18 years and older). Only adults were selected for the purpose of this review; MLTCs do occur in children, albeit at a lower prevalence.^25^

Participants also had to have MLTCs classified by a list of conditions in the studies, and were receiving care either in a primary, hospital or community setting. For the purpose of this review, the National Institute for Health Research’s (NIHR) definition^26^ of MLTCs, ‘existence of two more long-term health conditions in a single individual’ was used. Any studies that used the term ‘co-morbidity’ in reference to a single index condition were excluded as these studies focused instead on one specific condition as opposed to MLTCs.

Studies for inclusion consisted of quantitative studies only. As ethnicity was the primary focus of the review, only studies from North America or Europe were included as the majority of trials worldwide outside of these areas were likely to be carried out in an ethnically homogenous population. Any intervention with a main aim to prevent or manage the burden associated with multiple long-term health conditions was included.

Initial title and abstract screening against the eligibility criteria was carried out by two reviewers, ZK and SSO, via the online screening platform, Rayyan. Searches were updated and SJ conducted title and abstract screening for half of the articles between August 2022 and July 2023. Full-text screening of 152 articles was carried out by ZK, followed by SSO who screened 10% of the articles to assess level of agreement. No conflicts occurred, and there was 100% agreement between the two reviewers.

### Data extraction

The data extraction process was guided by Krishnan et al.’s framework^27^ (Supplementary materials, Appendix 3). The framework provides a guide to reporting outcomes on ethnic minority representation in cohort studies and subsequently, the outcomes were adapted to include SES. ZK completed all the data extraction using a standardised data extraction form, with AW double extracting 10% of the total studies to ensure accuracy. The percentage agreement was 95%. The two reviewers resolved any disagreements through discussion.

The following data were extracted from each included study: general information about the study (study authors, date, design, country); demographic information; ethnicity reporting; and SES reporting.

### Risk of bias assessment

The Effective Public Health Practice Project Quality Assessment tool^28^ was used to assess risk of bias. The following domains are included: selection bias; study design; confounders; blinding; data collection methods; withdrawal/dropouts; intervention integrity; and analyses. In line with the instructions for using the quality assessment tool,^28^ reviewers scored each individual domain for the study and then calculated an overall global rating from strong to weak. ZK independently assessed risk of bias, with AR and AW assessing 50% each of the studies. Percentage agreement was 74.4% across tool items. Disagreements were resolved by discussion between the reviewers. Risk of bias data can be found in the supplementary materials, Appendix 4.

### Data synthesis

A narrative synthesis of the findings related to ethnicity and SES was undertaken due to the heterogeneity of data in the studies. The outcomes (ethnic groups reported, SES measures) were variable. A summary of ethnicity data is presented in a tabular format. Ethnicity-related data for each study comprised of descriptive statistics relating to ethnic groups (*n* and %) in intervention and control groups. Studies that included ethnicity data were compared with UK 2021 ONS statistics or United States of America 2020 census data. Descriptive statistics for SES-related data were also presented in a table.

## Results

**Figure 1.**
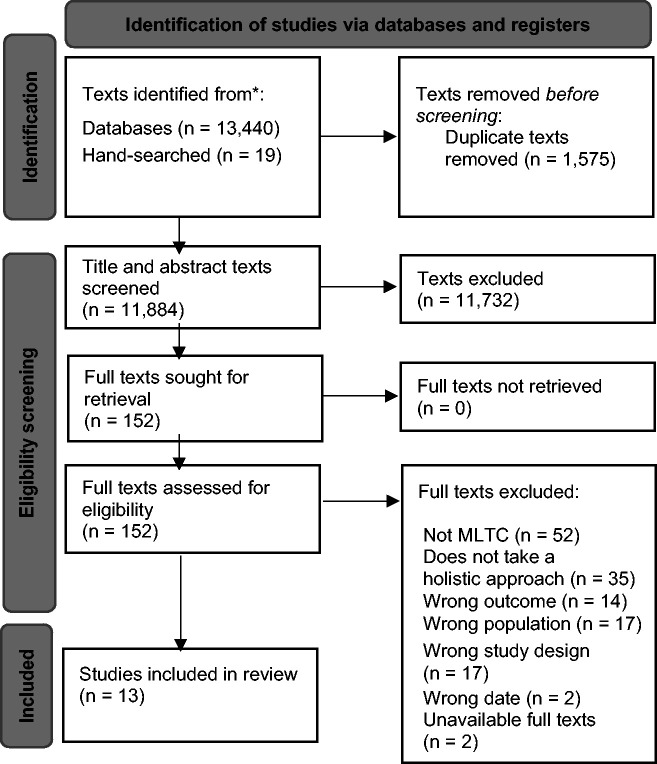
PRISMA (Preferred Reporting Items for Systematic reviews and Meta-Analyses) flowchart of the literature search.

A total of 11,884 articles were returned from searches once duplicates had been removed. A total of 152 full texts were screened. Overall, 13 articles met the inclusion criteria for this review (Figure 1).

### Study design

For this review, 12 randomised controlled trials (RCTs) and 1 before-and-after study design met the inclusion criteria.

### Participants

A total of 4,146 participants were included in the review (Table 1). Sample size ranged from 42 participants^29^ to 1546 participants.^30^ Five studies were based in Canada,^31–35^ two in the USA,^29,36^ two in Europe,^37,38^and four in the UK.^30,39–41^

**Table 1.** Characteristics of included studies.

Study	Study design	Participants	MLTCs classification and types	Intervention	Primary outcome	Risk of bias global rating
Contant et al.^31^	RCT	Canada,281 participants, aged between 18 and 75 years *(Mean = 53.5 years, SD = 11.2)* Men – 141 (50%)	At least three or more MLTCs, e.g. diabetes, COPD, CVD, asthma	Patient liaised with a nurse to develop a personalised intervention plan to manage their health conditions. The plan involved the input of other healthcare professionals in the field of nursing, nutrition, physical activity etc. Intervention consisted of at least three encounters with the HCPs	Health Education Impact Questionnaire (heiQ)	STRONG
del-Cura Gonzalez, 2022^37^	RCT	Spain593 participants aged between 65 and 74 years *(Mean = 69.7 years, SD = 2.7)* *Men – 262 (44%)*	At least three long-term chronic conditions	Patients collaborated with a physician to set negotiated treatment goals relating to the patients’ health conditions by taking into account their preferences. A treatment plan was then developed	Appropriateness of prescribing (Medication Appropriateness Index)	WEAK
Dunbar et al.^36^	RCT	USA134 participants aged between 29 and 81 years (*Mean = 57.4 years, SD – 10.6*) Men – 88 (65.7%)	At least two MLTCs, heart failure and diabetes	Patient educational sessions focused on diet, self-care, medications and symptom monitoring. Written materials were provided ‘HF-diabetes’ toolkit to be used at home. Phone counselling was also provided as well as home visits by a nurse	Quality of Life (EQ-5D)	STRONG
Fisher et al.^32^	RCT	Canada, 59 participants aged 65+ yearsMen – 30 (51%)	At least three or more MLTCs, e.g. diabetes, CVD, arthritis	Home visit by interdisciplinary team and support worker where they worked with patients and families to set goals in relation to patients needs and preferences for managing their conditions effectively. Monthly case conference meetings to discuss patients progress. Case management involved facilitating patients' access to appropriate healthcare services	Physical component score (SF-12)	WEAK
Fortin et al.^33^	RCT	Canada 284 participants aged between 18 and 80 years *(Mean = 60.9 years, SD = 10.6)* Men *– 132 (46%)*	At least three or more MLTCs, e.g. hypertension, depression, diabetes, cancer, CKD	Healthcare professionals delivered self-management support to patients. Patients had a contact nurse who developed a goal care plan. Patients were then referred to the most appropriate HCP who matched patient goals, including referral to the nurse themselves	Self-management (Health education impact questionnaire) Self-efficacy for managing chronic diseases	WEAK
Jäger et al.^38^	RCT	Germany 273 participants who were 50+ years *(Mean = 72.2 years, SD = 8.9)* Men – 121 (44%)	At least three MLTCs e.g. heart disease, liver disease, cancer, anxiety	Structured medication counselling and review with GP. Patients received educational materials and reminders relating to medication adherence	Implementation (Brown Bag, medication review)	STRONG
Khunti et al.^41^	RCT	UK 353 participants aged between 40 and 95 years old (*Mean* = 67.8 years, *SD* = 9.38) Men – 161 (46%)	Two or more MLTCs, e.g. asthma, stroke, depression, diabetes, chronic kidney disease	Self-monitoring and goal setting delivered by a trained facilitator, focused on increasing physical activity and addressing non-disease specific self-management challenges, managing treatments and healthcare communication	Change in volume of daily physical activity, measured via accelerometer	STRONG
Markle-Reid et al.^34^	RCT	Canada 159 participants who were 65+ years Men – 70 (44%)	At least three MLTCs e.g. Diabetes, hypertension, CVD	Up to three home visits from nurse/dietician, monthly wellness group programme and ongoing nurse-led care coordination. Self-efficacy, self-management and holistic care were the key aspects for the management of the patients conditions	Quality of Life – Physical component summary	MODERATE
Mercer et al.^39^	RCT	UK 152 participants, aged between 30 and 65 years *(Mean = 52 years)* Men – 67 (44%)	At least two or more MLTCs	Organisational-based intervention, longer structured consultations, holistic assessment, care plan, self-support materials, e.g. mindfulness, CBT self-help	Quality of Life (EQ-5D-5L) and Wellbeing (W-BQ12)	MODERATE
Miklavcic et al.^35^	RCT	Canada 132 participants, aged 65+ yearsMen – 60 (45%)	Two or more MLTCs, e.g. Depression, stroke, asthma, CVD	Three home visits by nurse/dietician, monthly group wellness programme including educational materials and peer support, care coordination, monthly case conferences	Physical functioning (SF-12)	STRONG
O’Toole et al.^40^	RCT	UK 149 participants with a mean age of 65.5 years (*SD* = 9.3) Men – 46 (31%)	Two or more MLTCs	Educational group-based programme focusing on diet, exercise, sleep, medication. Goal setting related to MLTCs management priorities	Health-related Quality of Life (EQ-5D-3L) Frequency of activity participation (Frenchay activities index)	WEAK
Salisbury et al.^30^	RCT	UK 1546 participants aged 18+ years (*Mean* = 70.9 years) Men – 763 (49%)	At least three MLTCse.g. stroke, epilepsy, COPD, depression	3D patient-centred reviews, nurse (health checks, depression screening, health promotion), pharmacist (inappropriate drugs, simplify medicine regimen), physician (managing conditions goals, timelines, plan)	QoL (EQ-5D-5DL)	STRONG
Watkins et al.^29^	Before and after study	USA 42 participants with a mean age of 55.9 years (SD = 9.5)	At least two MLTCse.g. hypertension, diabetes, hyperlipidaemia, depression	Pharmacist-driven health coaching (specialised advice, medication adherence, lifestyle management, sleep, smoking cessation)	Clinical outcomes (SBP, DBP, HbA1c)	WEAK

COPD: chronic obstructive pulmonary disease; CVD: cardiovascular disease; HCP: healthcare professional; RCT: randomised controlled trial; MLTCs: multiple long-term conditions; CKD: chronic kidney disease; GP: general practitioner; CBT: cognitive behavioural therapy.

Seven out of 13 studies focused on patients who were aged 50 years or above, while the remaining six studies^30,31,33,36,39,41^ incorporated a wider age range. All studies focused on a range of different long-term health conditions, including diabetes, cardiovascular diseases, depression, anxiety, arthritis and asthma. The criteria for MLTCs in the studies were clearly defined. In line with the NIHR’s definition for MLTCs, participants were required to have at least two long-term conditions, although some studies stated at least three long-term conditions^30,32–34,37,38,40^ as part of the inclusion criteria.

### Types of interventions

The types of interventions were classified using the Cochrane Effective Practice and Organisation of Care (EPOC, 2002)^42^ which has previously been used in Smith et al.’s systematic review of MLTCs interventions and outcomes.^23^ This taxonomy provides definitions for the types of interventions that are used in studies that explore more than one chronic condition. Interventions are classified into four categories: professional interventions aimed at changing clinicians' behaviour; financial interventions that offer providers with an incentive for reaching targets; patient-oriented interventions that focus on self-management support for patients, including educational support; and organisational interventions that change or alter care delivery.

The included studies in this review were patient-oriented interventions and organisational interventions. Out of the 13 studies, 6 (46%) were identified as being patient-oriented^31,33,34,36,39,40^ and 7 (54%) were identified as organisational-based interventions.^29,30,32,35,37,38^

### Primary study outcomes assessed

Studies had varying aims and associated outcomes. Quality of life,^30,34,36,39,40^ physical functioning,^32,35,41^ clinical outcomes measures (e.g. systolic blood pressure),^29^ medication review relating to the appropriateness of prescribing^37,38^ and impact of health education on self-management of MLTCs^31,33^ were primary outcomes identified.

### Risk of bias

Overall, the included studies had varying risk of biases ranging from strong to weak (Table 1).

Six out of 13 studies (46%)^30,31,35,36,38,41^ were strong, two were moderate (15%)^34,39^ and five were weak (39%).^29,32,33,37,40^

Most of the studies were particularly strong at reporting withdrawals or drop-outs. However, blinding of participants was not frequently conducted.

### Reporting and representation of ethnicity

Out of the 13 included studies, 4 (31%)^30,33,36,41^ reported on the proportion of participants by ethnic group (Table 2). The remaining nine studies did not report the ethnicity of participants. Of the four studies that reported information on ethnicity, three studies^30,33,41^ were made up of predominantly White participants and one study^36^ incorporated 69.4% of African American participants. In comparison with US 2020 census data,^43^ only 12.4% of the population identified as Black or African American; therefore, in this study, Black African American participants were overrepresented. Furthermore, only Dunbar et al.’s^36^ study took ethnicity into account in the analysis, by including ethnicity among other covariates to estimate treatment effects.

**Table 2.** Reporting and representation of ethnicity in included studies.

Study	Was ethnicity reported?	Method of reporting	Ethnic group coding	Breakdown of ethnic groups – Intervention *n* (%)	Breakdown of ethnic groups – Control *n* (%)	Breakdown of ethnic groups – Total *n*(%)	Ethnicity taken into account in analysis?	Cultural adaptations/tailoring?	Selection bias
Contant et al.^31^	No	NA	NA	NA	NA	NA	NA	NA	NA
del-Cura Gonzalez, 2022^37^	No	NA	NA	NA	NA	NA	NA	NA	NA
Dunbar et al.^36^	Yes	Self-reported/Medical record	African American	52 (74.3%)	41 (64.1%)	93 (69.4%)	Y	No	English language fluency
Fisher et al.^32^	No	NA	NA	NA	NA	NA	NA	NA	Speak English or access to translator
Fortin et al.^33^	Yes	Self-reported	White	100%	100%	100%	NA	No	Able to speak French, read, give consent
Jäger et al.^38^	No	NA	NA	NA	NA	NA	NA	NA	NA
Khunti et al.^41^	Yes	Self-reported	White, South Asian, Other	White 173 (96.1%)South Asian 6 (3.3%)Other 1 (0.6%)	White 168 (97.1%)South Asian 4 (2.3%)Other 1 (0.6%)	White 342 (96.6%)South Asian 10 (2.8%)Other 2 (0.6%)	No	No	Good understanding of English
Markle-Reid et al.^34^	No	NA	NA	NA	NA	NA	NA	NA	Speak English/access to translator
Mercer et al.^39^	No	NA	NA	NA	NA	NA	NA	NA	Excluded if they could not understand spoken/written English
Miklavcic et al.^35^	No	NA	NA	NA	NA	NA	NA	NA	Speak English
O’Toole et al.^40^	No	NA	NA	NA	NA	NA	NA	NA	NA
Salisbury et al.^30^	Yes	Self-reported	White, Other/Unknown	White 775 (97%) Other/Unknown 22 (3%)	White 729 (97%) Other/Unknown 20 (3%)	NA	No	No	Ability to complete questionnaires in English
Watkins et al.^29^	No	NA	NA	NA	NA	NA	NA	NA	NA

NA: Not applicable.

**Table 3.** Reporting and representation of SES in included studies.

Study	SES reported?	How was SES measured?	Method of reporting	SES breakdown – Intervention (*n*)%	SES breakdown – Control (n)%	SES breakdown - Total (*n*)%	SES accounted for in analysis?	SES selection bias
Contant et al.^31^	Yes	Family income ($), education, perceived financial status	Self-reported	Income: <$20,000 (19) 14% $20,000–49,999 (51) 37%≥$50,000 (65) 47%Education: High school degree or less (65) 47%Technical, college or university (72) 52% Financial status: Poor/really poor (9) 6%Enough (76) 55%Well off (44) 32%	Income: <$20,000 (17) 12% $20,000–49,999 (56) 39%≥$50,000 (66) 46%Education: High school degree or less (80) 56%Technical, college or university (62) 44% Financial status: Poor/really poor (16) 11%Enough (79) 56%Well off (22) 15%		Yes	No
del-Cura Gonzalez, 2022^37^	Yes	Education, employment, monthly income	Self-reported	Education: Not completed primary (137) 46%Completed primary (92) 30.9% Bachelor or higher (69) 23.2%Occupation: Supervisor, middle management and director (121) 40.6%Skilled primary sector (109) 36.6% Unskilled (68) 22.8% Monthly income: ≤1050 euro (82) 27.5% 1051–2250 euro (179) 60.1%≥2251 euro 30) 10.1% Unknown (7) 2.3%	Education: Not completed primary (142) 48.1%Completed primary (104) 35.3%Bachelor or higher (49) 16.6% Occupation: Supervisor, middle management and director (113) 38.3%Skilled primary sector (108) 36.6%Unskilled (74) 25.1% Monthly income: ≤1050 euro (88) 29.8% 1051–2250 euro (163) 55.3%≥2251 euro (29) 9.8%Unknown (15) 5.1%	Education: Not completed primary (279) 47.0%Completed primary (196) 33.1% Bachelor or higher (118) 19.9%Occupation: Supervisor, middle management and director (234) 39.5%Skilled primary sector (217) 36.6%Unskilled (142) 23.9%Monthly income: ≤1050 euro (170) 28.7%1051–2250 euro (342) 57.7% ≥2251 euro (59) 9.9%Unknown (22) 3.7%	No	No
Dunbar et al.^36^	Yes	Education	Self-reported	High school or less: (25) 35.7%	High school or less: (25) 39.1%	High school or less (50) 37.3%	No	No
Fisher et al.^32^	Yes	Annual income	Self-reported	Income: $0 to $39,999 (15) 50%$40,000+ (3) 10% Missing (12) 40%	Income: $0 to $39,999 (12) 41% $40,000+ (3) 10% Missing (14) 48%		No	No
Fortin et al.^33^	Yes	Education, household income, employment status	Self-reported	Education: Incomplete secondary school (36) 25%Completed secondary school (30) 20.8%College (66) 45.8%University (12) 8.3% Household income (CAD $) <20,000 (26) 18.1% 20,000–49,999 (52) 36.1% ≥50,000 (59) 41%Missing data (7) 4.9%Employment: Employed (51) 35.4%Unemployed (26) 18.1% Retired (67) 46.5%	Education: Incomplete secondary school (30) 21.4%Completed secondary school (38) 27.1%College (54) 38.6% University (18) 12.9% Household income (CAD $) <20,000 (26) 18.6%20,000–49,999 (54) 38.6% ≥50,000 (55) 39.3%Missing data (5) 3.6%Employment: Employed (45) 32.1%Unemployed (26) 18.6% Retired (69) 49.3%		No	No
Jäger et al.^38^	Yes	Employment, education	Self-reported	Not working (122) 85.9%Graduation from high school or university (7) 4.9%	Not working (110) 88.7% Graduation from high school or university (6) 4.6%	Not working (232) 87.2%Graduation from high school or university (13) 4.8%	No	One region in South Germany
Khunti et al.^41^	Yes	Employment status	Self-reported	Full/part-time employed 54 (30%) Retired 113 (62.8%) Unemployed/sick/disabled 13 (7.2%)	Full/part-time employed 46 (26.6%) Retired 112 (64.7%) Unemployed/sick/disabled 15 (8.7%)	Full/part-time employed 101 (28.6%)Retired 225 (63.7%) Unemployed/sick/disabled 27 (7.7%)	No	No
Markle-Reid et al.^34^	Yes	Annual income (CAD$)	Self-reported	CAD $ <40,000 – (36) 45.0%≥40,000 – (30) 37.5%Missing – (14) 17.5%	<40,000 – (31) 39.2% ≥40,000 – (29) 36.7% Missing – (19) 24.1%		No	No
Mercer et al.^39^	Yes	Index of Multiple Deprivation	Self-reported	Q1 (least deprived) (2) 3% Q2 (3) 4% Q3 (5) 6% Q4 (8) 11% Q5 (most deprived) (57) 76%	Q1 (least deprived) (2) 3% Q2 (1) 1% Q3 (4) 5% Q4 (7) 9% Q5 (most deprived), (61) 82%		No	Deprived areas in Glasgow
Miklavcic et al.^35^	Yes	Annual Income (CAD$)	Self-reported	$0 to $39,999, (17) 33.3%$40,000+, (34) 66.7%	$0 to $39,999 (26) 50.0%$40,000+ (26) 50.0%		No	No
O’Toole et al.^40^	Yes	Educational level and employment status	Self-reported	Educational level: Primary (29) 37.2%Some secondary (19) 24.4% Complete secondary (14) 17.9% College/university (16) 20.5% Employment status: Full-time employment (0) 0.0% Part-time employment (6) 7.7% Not working due to condition (17) 21.8%Unemployed (5) 6.4%Retired (46) 59.0%Carer (1) 1.3% Full-time housewife (3) 3.8%	Educational level: Primary (27) 38.0%Some secondary (20) 28.2%Complete secondary (11) 15.5% College/University (13) 18.3%Employment status: Full-time employment (2) 2.8% Part-time employment (1) 1.4% Not working due to condition (23) 32.4% Unemployed (5) 7.0% Retired (37) 52.1%Carer (2) 2.8%Full-time housewife (1) 1.4%		No	No
Salisbury et al.^30^	Yes	Employment status	Self-reported	Fully retired (525) 66%Other/unknown occupational status (272) 34%	Fully retired (512) 68%Other/unknown occupational status (237) 32%	N/A	No	Deprivation status, GP practices
Watkins et al.^29^	No	NA	NA	NA	NA	NA	NA	NA

NA: Not applicable; SES: socioeconomic status; GP, general practitioner.

All information relating to ethnic groups was self-reported by participants. Fortin et al.^33^ clearly stated that all participants were White, and similarly, Salisbury et al.^30^ described groups as either ‘White’ or ‘Other/Unknown’ in which 97% of total participants were White, and 3% identified as Other/Unknown. White participants were overrepresented in Salisbury et al.’s study when compared with UK census data.^44^ Ethnic minority groups were underrepresented in the study.

Similarly, in Khunti et al.’s ^41^ study, 96.6% of the total sample were White. In the study, 2.8% of the sample identified as ‘South Asian’ and 0.57% as ‘Other’, whereas UK census data from 2021^44^ show that 9.3% of the population belongs to the Asian ethnic group. No information was provided on South Asian subgroups and those participants who identified as ‘Other’.

In 8 of the 13 studies, there were selection biases whereby the inclusion criteria explicitly stated that participants must be able to speak English (or the country’s national language) or have access to a translator. No studies reported any cultural adaptations or tailoring, such as the use of translators or translated materials.

### Reporting and representation of SES

Altogether, 12/13 (92%) of studies reported information on SES, which was self-reported by participants. Income and/or educational level were the most common methods of measuring SES.^31,32,34–38,40^ Six studies also included participants’ employment status to measure SES.^30,33,37,38,40,41^ One study^31^ measured participants' self-perceived financial status while another used an index of multiple deprivation to determine participants' SES.^39^

The spread of SES groups within study samples was varied. In Jäger et al.’s study,^38^ 85.9% of participants in the intervention group were not working and only 4.9% had graduated from high school or university. The sample^38^ consisted mainly of higher deprivation groups based on findings from educational attainment and employment status alone. O’Toole et al.^40^ identified 59% of participants as retired in the intervention group as a category for employment status, but no data were collected on pre-retirement occupation. Likewise, Khunti et al.^41^ reported that 62.8% of the intervention group were retired. No further information was provided with regard to participants’ pre-retirement occupations.

Studies differed in their representation of SES groups based on income. In Contant et al.’s study,^31^ 14% of participants in the intervention had an income of under C$20,000, with the majority (47%) reporting an income of more than C$50,000. Similarly, Fortin et al.’s study^33^ included 18.1% of participants in the intervention group with an income of under C$20,000, and 41% of participants with an income that was equal to or more than C$50,000. In comparison to the Canadian population, findings for the lower income brackets align with Canadian census data.^45^ The percentage of the Canadian population in the higher income bracket, C$50,000 to C$59,999, was 9.7%. In both studies,^31,33^ participants in the higher income bracket were overrepresented when compared with the general population.

Across the six studies that measured income, two studies had a higher proportion of low SES groups.^32,34^ In Fisher et al.’s^32^ study, 50% of participants had an income between $0 and $39,999, and Markle-Reid et al.’s^34^ study included 45% of participants who had an income under CAD$40,000.

Three studies^30,38,39^ had selection biases relating to the geographical region where participants were recruited from. For example, Jäger et al.’s^38^ study recruited from one geographical region in South Germany only.

## Discussion

### Summary

A total of 13 studies were identified for inclusion in this review. All studies were RCTs, except one that was a before-and-after study. Despite the limited number of eligible studies, it is clear that ethnicity is underreported and ethnic minority groups are underrepresented in studies of people with MLTCs, whereas SES was reported in the majority of the studies but there were issues relating to representation of SES groups due to limited data. Under- or overrepresentation of either higher or lower SES groups may fail to capture the socioeconomic diversity of participants, reducing the validity of studies. Regarding ethnicity reporting, only 4 out of 13 (31%) studies reported information on ethnicity, including all ethnic groups studied. SES was commonly reported in the studies, using a variety of different indicators. Twelve out of 13 studies (92%) reported SES and provided information on socioeconomic groups through a variety of measures.

### Comparisons with previous studies

These findings mirror the underreporting of ethnicity across health and care research.^46^ A systematic review of cardiovascular trials found that only 140 out of 250 (56%) RCTs included reported on race/ethnicity.^47^ Additionally, a study reviewing trial documentation for 407 RCTs reported that ethnicity was recorded in 67.3% of trials. Only 9.3% of trials accurately detailed the methods used for recording and reporting ethnicity.^48^

Given the current awareness regarding this topic in recent years,^30^ the results are concerning. Most of the inclusion criteria specified that participants had to speak or have a good understanding of a certain language (usually English), and no attempts were made to provide translators or other cultural adaptations in any of the studies. Limiting cultural adaptations of MLTCs management/treatment and the poor recruitment of ethnic minority groups to evaluation studies could limit the development of effective interventions for these groups.

In contrast to previous findings in the literature,^49,50^ many of the studies included in this review had reported SES. Some studies mainly included older adults as they are more likely to have MLTCs; however, when determining employment status for these participants, they were usually retired. For retired participants, collecting data on their former occupation can be an indicator of their current lifestyle and in turn, their social status. For older adults, data should ideally be collected on the transition from working to retirement age as this aligns with socioeconomic differences between groups.^51,52^ The lack of detailed reporting on SES, in relation to pre-retirement occupations and the use of only one or two SES measures made it challenging to determine how well low SES groups are represented.

### Strengths and limitations

To our knowledge, this review is the first review to report on the reporting and representation of ethnicity and low SES in intervention studies for MLTCs. A comprehensive set of databases (MEDLINE, Scopus, Cochrane Library and CINAHL) were searched for relevant studies. The review adheres to key guidelines^22^ on the development, conduct and reporting of systematic reviews. Only studies from Europe and North America were included as the majority population in these continents is White, and therefore what constitutes minority status in these regions differs from other continents, making it problematic to synthesise findings. Furthermore, there is less diversity and more ethnic homogeneity in the regions not included in this review.

There were a small number of studies included in this review. A meta-analysis could not be conducted due to the limited ethnicity-related data retrieved from studies. Similarly, for SES related data, the heterogeneity of the different indicators used meant that the data were not appropriate for pooling. Having sufficient data to conduct a meta-analysis would have been advantageous as this would have provided an indication of the overall proportion of ethnic minority and SES representation across the evidence base. MLTCs was the focus for this review as opposed to ‘co-morbidities’, which is often used in reference to a single index condition.^53^ This approach aligns with recent systematic reviews^23,24^ on MLTC interventions. While this may have excluded some of the older research studies, systematic reviews were hand searched instead for interventions potentially meeting the inclusion criteria.^23,24^ The search process may also have been limited by ‘MLTCs’ not being identified as a MeSH term by the databases used.

### Implications of findings and future directions

The information retrieved from the studies and summarised in this systematic review is of great importance to researchers and clinicians alike, as it contributes towards a growing evidence base in this field. It is important to understand which groups of people are participating in health research and who is underrepresented. Consistently reporting information on participants’ ethnicity and SES in studies is vital. Researchers can draw on the findings from this review to ensure that their research, especially the inclusion criteria, is representative of target populations and allows for greater participation of underserved groups of whom research might be of most benefit. Tools are available, such as the Equality Impact Assessment Toolkit (EqIA),^54^ which ensures that those at a disadvantage or underrepresented are visible in research settings and that their needs are being met. Recently, the UK Health Data Research Alliance report (2023)^55^ highlighted the importance of ethnicity data collection for clinicians, researchers and policy makers in order to improve understanding of diseases and management of care for ethnic minority groups. The report emphasised that a national framework for ethnicity data collection should be implemented and recommendations were made. These included the need for ethnicity data as well as SES data to be gathered consistently across healthcare sectors, ethnicity coding to be standardised in line with Office for National Statistics categories and training healthcare professionals to understand the importance of this data collection.^55^

Some long-term conditions like type 2 diabetes and cardiovascular diseases affect certain ethnic groups more than others.^56,57^ UK South Asians are six times more likely than their White British counterparts to have type 2 diabetes^57^; however, they are underrepresented in diabetes trials. A study on cardiovascular outcome trials of glucose-lowering therapies in type 2 diabetes found that South Asian participants were underrepresented when compared with the UK and global diabetes population.^59^ Reporting of ethnicity within trials was also not optimal as studies failed to provide a breakdown of specific South Asian sub-groups.^59^ Thus, these groups should be consistently participating in research for interventions to be developed, which are culturally competent.

There is a need for research guidance as part of best practice to include and standardise reporting data on participants' ethnicity and SES. These data should be recognised as being equally as important as reporting participants’ sex and age. Better representation of underserved groups is needed in health research.^60^ This would contribute towards reducing any potential health inequalities and would ensure health research is reflective of those groups who it may be most advantageous for.

## Conclusions

The findings from this review indicate that there is a lack of reporting on ethnicity and underrepresentation of ethnic minority groups in MLTCs intervention studies; however, SES is well reported but representation varies across the spectrum of SES. Future MLTC intervention studies should focus on improving the recruitment of ethnic minority groups in particular, and ensure they report on the ethnicity of included samples. Low SES groups should also be consistently represented in MLTC intervention studies and effort should be made to improve recruitment of these groups as studies of interventions may benefit these specific groups the most.

## Supplemental Material

sj-pdf-1-jrs-10.1177_01410768241233109 - Supplemental material for Reporting and representation of underserved groups in intervention studies for patients with multiple long-term conditions: a systematic review Supplemental material, sj-pdf-1-jrs-10.1177_01410768241233109 for Reporting and representation of underserved groups in intervention studies for patients with multiple long-term conditions: a systematic review by Zara Kayani, Andrew Willis, Shukrat O Salisu-Olatunji, Shavez Jeffers, Kamlesh Khunti and Ash Routen in Journal of the Royal Society of Medicine

## Declarations

### Guarantor

ZK.

### Provenance

Not commissioned; peer-reviewed by Jonathan Valabhji and Julie Morris.
